# Is extension of multi-level lumbar fusion to S1 necessary in the absence of L5–S1 instability? A comparative study of selective decompression versus TLIF with S1 pedicle screw fixation

**DOI:** 10.3389/fsurg.2026.1874165

**Published:** 2026-07-29

**Authors:** Ahmet Kürşat Kara, Zahir Kızılay

**Affiliations:** 1 Department of Neurosurgery, Antalya City Hospital, Antalya, Türkiye; 2 Department of Neurosurgery, Faculty of Medicine, Aydın Adnan Menderes University, Aydın, Türkiye

**Keywords:** decompression, L5–S1, lumbar fusion, S1 pedicle screw, sacral fixation, screw loosening, spinal instability, TLIF

## Abstract

**Background:**

S1 pedicle screw loosening remains a clinically significant complication in lumbosacral fusion, with reported incidences ranging from 15.6% to 41.9%. In patients undergoing multi-level posterior lumbar fusion with concomitant symptomatic but stable L5–S1 pathology, it remains unclear whether extending the construct to the sacrum is necessary.

**Methods:**

This retrospective study included 60 patients who underwent multi-level posterior lumbar instrumentation (fusion constructs spanning 2–4 segments) between November 2023 and April 2024. All patients had symptomatic L5–S1 pathology (disc herniation or stenosis) without radiographic instability, and required fusion for upper lumbar pathology. Patients with L5–S1 instability (>3 mm translation or >10° angulation on flexion–extension radiographs) were excluded. Group 1 (*n* = 30) underwent selective L5–S1 decompression without S1 fixation; Group 2 (*n* = 30) underwent TLIF with S1 pedicle screw instrumentation. Clinical outcomes (ODI and VAS), perioperative parameters, and complications were evaluated at 1, 6, and 24 months.

**Results:**

Groups were comparable at baseline. Operative time (148.4 ± 26.1 vs. 204.7 ± 32.8 min, *p* < 0.001), estimated blood loss (376.7 ± 114.3 vs. 653.3 ± 211.8 mL, *p* < 0.001), and hospital stay (3.7 ± 0.9 vs. 4.4 ± 0.8 days, *p* = 0.004) were significantly lower in Group 1. Both groups demonstrated significant within-group improvements in ODI and VAS scores at all time points (*p* < 0.001). No statistically significant between-group differences were observed in ODI or VAS at 6 months or 24 months. Group 1 showed significantly lower VAS at 1 month (*p* = 0.022). Radiographic S1 screw loosening was observed in 7 of 30 patients (23.3%) in Group 2.

**Conclusion:**

In this retrospective cohort, selective decompression of the L5–S1 segment without S1 instrumentation was associated with comparable mid-term clinical outcomes while reducing operative time, blood loss, and S1 screw-related complications.

## Introduction

Despite advancements in surgical techniques and instrumentation, loosening of S1 screws remains a significant complication. In lumbar fusion surgery, this complication has been reported in various studies with an incidence ranging from 15.6% to 41.9% (1).

The higher rate of loosening in S1 screws compared to lumbar screws is generally attributed to the increased mechanical load in this region, as well as the anatomical characteristics of S1 pedicles, which contain a higher proportion of cancellous bone and are relatively shorter and wider (2, 3).

In recent years, pedicle screw fixation has been increasingly utilized as a standard surgical stabilization technique in the management of spinal disorders, including lumbar stenosis, degenerative disc disease, spondylolisthesis, and scoliosis (4).

Several approaches have been explored to decrease the risk of S1 screw loosening, such as performing interbody fusion at the L5–S1 level and placing S1 screws in a bicortical manner. However, the use of interbody fusion may be associated with longer operative time, higher complication rates, and increased healthcare costs (5, 6).

In clinical practice, patients undergoing multi-level posterior lumbar stabilization frequently present with concomitant symptomatic L5–S1 pathology such as disc herniation or foraminal stenosis causing radicular pain and/or neurological deficits, yet without radiographic evidence of segmental instability at this level. In such cases, the decision of whether to extend the fusion construct to the sacrum or to perform selective decompression of the L5–S1 segment without S1 pedicle screw fixation remains a matter of ongoing clinical debate. The present study was designed to address this specific question by comparing perioperative and clinical outcomes between these two surgical strategies in a homogeneous cohort of patients without L5–S1 instability.

## Materials and methods

### Study design and patient selection

This retrospective study includes a review of medical records and radiological images of 60 patients operated on by our team at the Neurosurgery Department (BLINDED FOR REVIEW) between November 2023 and April 2024.

**Inclusion criteria:** (1) patients undergoing posterior instrumented lumbar fusion for degenerative pathology at one or more levels above L5–S1; (2) concomitant symptomatic L5–S1 pathology (disc herniation, foraminal stenosis, lateral recess stenosis, or mixed stenosis/disc) causing radicular pain and/or neurological deficits; (3) absence of L5–S1 segmental instability on preoperative flexion–extension radiographs; and (4) minimum follow-up of 24 months.

**Exclusion criteria:** (1) presence of L5–S1 instability on flexion–extension radiographs, defined as more than 3 mm of translation or more than 10° of angulation — these patients were excluded as sacral fixation is mandatory in such cases and meaningful comparison is not possible; (2) absence of any suspected pathology at the L5–S1 level; (3) prior surgery at the lumbosacral junction; (4) pathology of traumatic, infectious, or neoplastic etiology.

Patients meeting all inclusion criteria were assigned to one of two surgical groups based on preoperative assessment of the feasibility of facet-preserving decompression at the L5–S1 level, with final confirmation intraoperatively. For each patient, preoperative MRI findings and clinical presentation were evaluated to determine whether adequate neural decompression could be achieved without violating the facet joint. In patients where facet-preserving decompression was deemed feasible, selective foraminotomy and/or discectomy was performed without sacral instrumentation (Group 1). In patients where the extent of pathology precluded facet preservation and necessitated facetectomy, TLIF with bilateral S1 pedicle screw fixation was performed, as facetectomy inherently compromises segmental stability and mandates instrumented fusion (Group 2). In cases where intraoperative findings unexpectedly precluded facet preservation, TLIF with S1 fixation was performed accordingly. Group 1 consisted of patients in whom selective decompression (foraminotomy and/or discectomy) was performed at L5–S1 without extending the fusion construct to the sacrum. Group 2 consisted of patients in whom the fusion was extended to S1 with TLIF and bilateral S1 pedicle screw fixation.

### Radiological evaluation

All patients underwent a standardized preoperative radiological assessment including standing anteroposterior and lateral lumbar radiographs, dynamic flexion–extension lateral radiographs, and lumbar MRI. Lumbar CT was additionally evaluated when available, particularly for the assessment of bony foraminal stenosis, facet joint morphology, screw position, and postoperative fusion-related findings.

Dynamic instability at the L5–S1 level was assessed using flexion–extension lateral radiographs. Sagittal translation was measured as the displacement between the posterior vertebral body lines of L5 and S1, and angular motion was measured as the change in the L5–S1 disc angle between flexion and extension views. L5–S1 instability was defined as more than 3 mm of sagittal translation or more than 10° of angular motion (7). Patients exceeding these thresholds were excluded from the study.

Preoperative MRI was used to determine the type and extent of L5–S1 pathology, including disc herniation, central canal stenosis, lateral recess stenosis, foraminal stenosis, and mixed stenosis/disc pathology. Sagittal and axial T2-weighted images were primarily used to evaluate neural compression, while sagittal T1- and T2-weighted images were used to assess disc degeneration, disc height loss, foraminal narrowing, perineural fat obliteration, and facet joint hypertrophy.

The choice between selective L5–S1 decompression without S1 fixation and L5–S1 TLIF with S1 pedicle screw fixation was based primarily on preoperative MRI and, when available, CT findings. The key preoperative consideration was whether adequate neural decompression could be achieved while preserving the majority of the L5–S1 facet joint and posterior stabilizing structures. Selective decompression without S1 fixation was planned when the L5–S1 pathology was considered amenable to limited foraminotomy, partial hemilaminectomy, medial facet undercutting, and/or targeted discectomy without the need for extensive facet joint removal. This approach was preferred in patients with soft disc herniation, lateral recess stenosis, or limited foraminal stenosis in whom adequate decompression could be achieved using facet-preserving techniques.

In contrast, extension of the construct to S1 with L5–S1 TLIF was planned when preoperative imaging suggested that sufficient decompression would require extensive facet joint resection, such as subtotal or complete facetectomy, or when severe foraminal stenosis was associated with marked disc height collapse requiring restoration of foraminal height with an interbody cage. Severe bony foraminal stenosis, marked facet hypertrophy, complete perineural fat obliteration around the exiting nerve root, and foraminal narrowing unlikely to be adequately decompressed with facet-preserving techniques were considered imaging features favoring TLIF with S1 fixation. The final surgical strategy was confirmed intraoperatively according to the extent of decompression required to achieve adequate neural decompression without creating iatrogenic instability.

Postoperative radiographs and/or CT images obtained during follow-up were reviewed to evaluate implant position, construct extent, fusion-related findings, L5–S1 segmental changes, and S1 pedicle screw loosening in patients who underwent sacral fixation. Radiographic S1 screw loosening was defined as the presence of a peri-screw radiolucent halo greater than 1 mm at the bone–implant interface and/or progressive change in screw position on serial imaging. L5–S1 pseudarthrosis was suspected in the presence of absent bridging bone, persistent radiolucency across the fusion bed, cage subsidence, or hardware-related failure on follow-up imaging.

**Figure 1:**
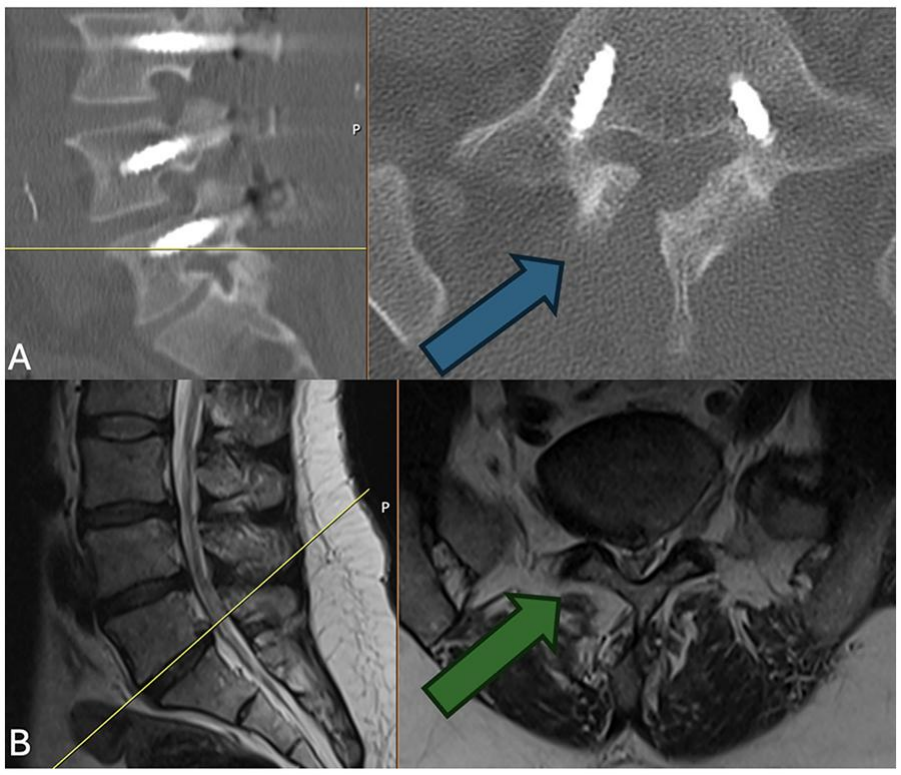
Representative group 1 case treated with selective L5–S1 decompression without S1 fixation. **(A)** Postoperative sagittal and axial CT images showing multi-level posterior instrumentation ending at L5, without extension to S1. The blue arrow demonstrates preservation of the L5–S1 facet joint/posterior elements after facet-preserving decompression. **(B)** Preoperative sagittal and axial T2-weighted MRI images of the same patient showing L5–S1 disc herniation with foraminal/lateral recess stenosis. The green arrow indicates the neural compression site. Because preoperative imaging suggested that adequate decompression could be achieved with limited foraminotomy and/or discectomy while preserving the majority of the facet joint, S1 fixation was not performed.

**Figure 2:**
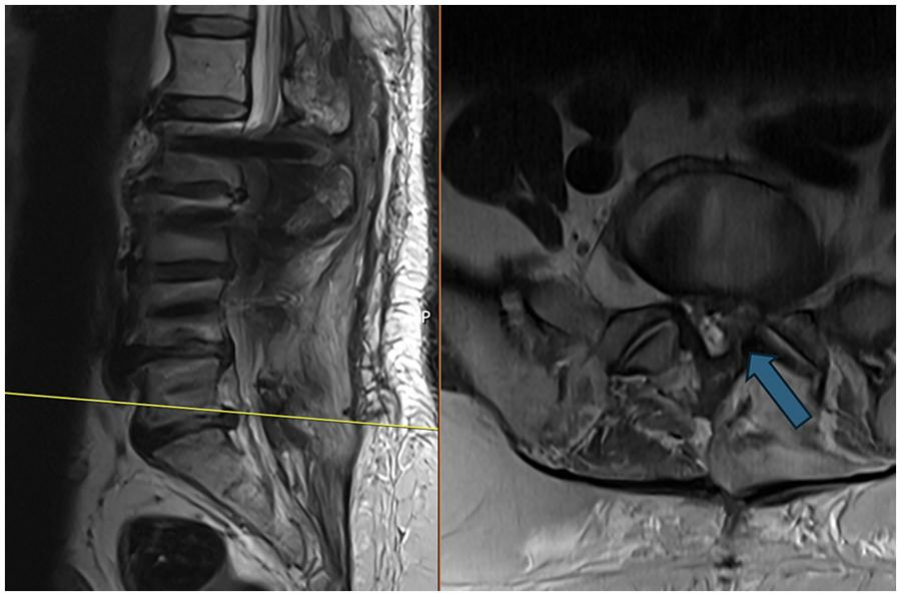
Representative late revision case in group 1 after selective decompression without S1 fixation. Postoperative sagittal and axial T2-weighted MRI obtained 18 months after selective L5–S1 decompression without S1 fixation demonstrating recurrent/progressive L5–S1 disc herniation with neural compression. The blue arrow indicates the recurrent disc fragment/compression site. The patient developed recurrent radicular symptoms and subsequently required revision surgery.

**Figure 3:**
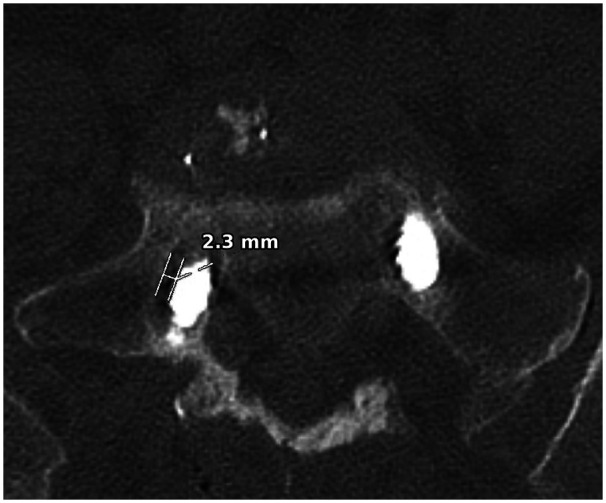
Representative example of S1 pedicle screw loosening in a Group 2 patient. Axial CT image demonstrates a peri-screw radiolucent halo measuring 2.3 mm at the bone-implant interface around the S1 pedicle screw, consistent with the study's radiographic definition of loosening (peri-screw halo > 1 mm).

**Figure 4:**
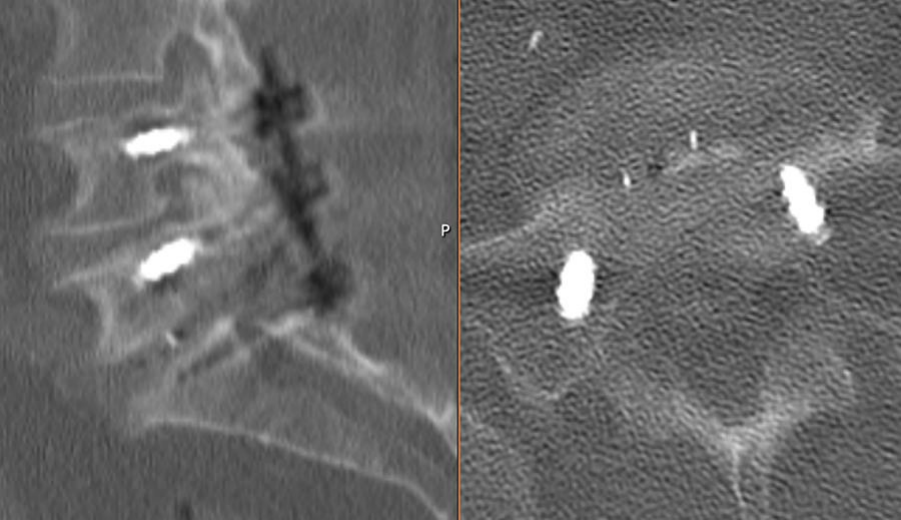
Representative group 2 case demonstrating a well-incorporated L5–S1 transforaminal lumbar interbody fusion (TLIF) with intact sacral fixation. Sagittal and axial CT reconstructions show the multi-level posterior instrumentation construct extending to S1, with solid bony bridging across the L5–S1 interbody space and bilateral S1 pedicle screws demonstrating a tight bone–screw interface without peri-screw lucency, consistent with stable fixation. TLIF, transforaminal lumbar interbody fusion; CT, computed tomography.

**Figure 5:**
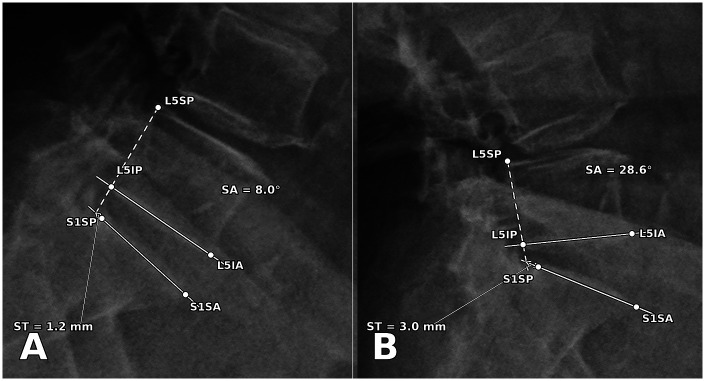
Representative excluded case demonstrating L5–S1 instability on flexion–extension radiographs. Lateral radiographs obtained in flexion **(A)** and extension **(B)** were used for dynamic assessment of the L5–S1 segment. The segmental angle (SA) was measured between the inferior endplate of L5 and the superior endplate of S1. Sagittal translation (ST) was measured as the displacement between the posterior wall of L5 (dashed line connecting L5SP and L5IP) and the posterosuperior corner of S1 (S1SP), at the level of the S1 superior endplate. In this representative case, SA increased from 8.0° in flexion to 28.6° in extension, corresponding to an angular motion of 20.6°, while ST was 1.2 mm in flexion and 3.0 mm in extension. Because angular motion exceeded the predefined 10° threshold, this case was considered radiographically unstable at L5–S1 and was excluded from the study cohort. L5SP, L5 superior posterior point; L5IP, L5 inferior posterior point; L5IA, L5 inferior anterior point; S1SP, S1 superior posterior point; S1SA, S1 superior anterior point; SA, segmental angle; ST, sagittal translation.

All radiological evaluations were independently reviewed by a neurosurgeon and a radiologist. Disagreements were resolved by consensus. Representative annotated imaging examples demonstrating the evaluation of L5–S1 stability, preoperative MRI-based surgical planning, facet-preserving decompression, TLIF with S1 fixation, and S1 screw loosening are provided in Figures 1–5.

### Surgical technique

All procedures were performed via a standard open posterior midline approach with conventional subperiosteal muscle dissection. In Group 1, facet joint integrity was preserved during surgery. In cases of disc herniation, foraminotomy was performed with partial hemilaminectomy and targeted discectomy when necessary. In cases of central compression due to ligamentum flavum hypertrophy or canal stenosis, conventional laminectomy was performed to achieve adequate decompression while preserving the facet joints. In Group 2, standard open TLIF was performed at the L5–S1 level with bilateral S1 pedicle screw fixation.

### Outcome measures

The primary clinical outcomes were ODI and VAS scores, assessed preoperatively and at postoperative months 1, 6, and 24. Perioperative outcomes including operative time, estimated blood loss, hospital stay, and transfusion requirement were also recorded. Complications including dural tear, wound complication, new neurological deficit, and early reoperation within 30 days were documented. During follow-up, revision surgery requirements and radiographic findings at the L5–S1 level were evaluated. In Group 2, radiographic S1 screw loosening, symptomatic loosening, and pseudarthrosis were specifically assessed.

### Statistical analysis

Statistical analyses were performed using IBM SPSS Statistics for Windows, Version 25.0 (IBM Corp., Armonk, NY, USA). Continuous variables are expressed as mean ± standard deviation (SD). The normality of distribution was assessed using the Shapiro–Wilk test. For between-group comparisons, the independent samples t-test was used for normally distributed variables, and the Mann–Whitney U test for non-normally distributed variables. Categorical variables were compared using the chi-square test or Fisher's exact test as appropriate. Fusion level distribution across groups was analyzed using the chi-square test. Within-group changes in ODI and VAS scores over time were assessed using the Wilcoxon signed-rank test. A two-tailed *p* value of < 0.05 was considered statistically significant. Preoperative lumbar lordosis was compared between groups using the independent samples t-test.

## Results

### Baseline characteristics

A total of 60 patients were included, with 30 in each group. The mean age was 61.1 ± 9.9 years in Group 1 and 59.3 ± 9.3 years in Group 2. Group 1 consisted of 14 females and 16 males; Group 2 consisted of 15 females and 15 males. The fusion construct in Group 1 extended from L4–L5 in 4 patients, L3–L5 in 14, and L2–L5 in 12. In Group 2, constructs extended to S1: L4–S1 in 5, L3–S1 in 11, and L2–S1 in 14 patients. No statistically significant differences were observed between groups in age, sex, BMI, fusion level distribution, or preoperative ODI and VAS scores (*p* > 0.05). The prevalence of diabetes mellitus (13.3% vs. 23.3%; *p* = 0.506), smoking (20.0% vs. 23.3%; *p* = 1.000), and osteoporosis or low bone mineral density (26.7% vs. 10.0%; *p* = 0.181) did not differ significantly between groups. Baseline characteristics are summarized in Table 1.

**Table 1:** Baseline demographic, clinical, and radiographic characteristics of the patients in both groups.

Variable	Group 1 (*n* = 30)	Group 2 (*n* = 30)	*p* value
Age (years)	61.1 ± 9.9	59.3 ± 9.3	0.474
Female/Male	14/16	15/15	1.000
BMI (kg/m^2^)	29.0 ± 3.0	30.1 ± 3.1	0.156
Diabetes mellitus, *n* (%)	4 (13.3%)	7 (23.3%)	0.506
Smoking, *n* (%)	6 (20.0%)	7 (23.3%)	1.000
Osteoporosis/low BMD, *n* (%)	8 (26.7%)	3 (10.0%)	0.181
Fusion levels			0.732
L4–L5/L4–S1	4 (13.3%)	5 (16.7%)	
L3–L5/L3–S1	14 (46.7%)	11 (36.7%)	
L2–L5/L2–S1	12 (40.0%)	14 (46.7%)	
Preoperative ODI	50.6 ± 6.8	49.0 ± 8.2	0.417
Preoperative VAS	7.5 ± 0.9	7.7 ± 1.0	0.551
Preoperative LL	41.8 ± 9.6	40.6 ± 10.2	0.642
L5–S1 pathology type			0.085
Disc herniation	4 (13.3%)	5 (16.7%)	
Foraminal stenosis	5 (16.7%)	8 (26.7%)	
Lateral recess stenosis	3 (10.0%)	6 (20.0%)	
Central canal stenosis	4 (13.3%)	7 (23.3%)	
Mixed stenosis/disc	14 (46.7%)	4 (13.3%)	

Values are expressed as mean ± SD or *n* (%).

ODI, Oswestry Disability Index; VAS, visual analogue scale; BMI, body mass index.

### Perioperative outcomes

Group 1 demonstrated significantly shorter operative time (148.4 ± 26.1 vs. 204.7 ± 32.8 min; *p* < 0.001), lower estimated blood loss (376.7 ± 114.3 vs. 653.3 ± 211.8 mL; *p* < 0.001), and shorter hospital stay (3.7 ± 0.9 vs. 4.4 ± 0.8 days; *p* = 0.004) compared to Group 2. Blood transfusion was required in 2 patients (6.7%) in Group 1 versus 8 patients (26.7%) in Group 2 (*p* = 0.080). Rates of incidental dural tear, wound complication, new neurological deficit, and early reoperation within 30 days did not differ significantly between groups. Perioperative outcomes are summarized in Table 2.

**Table 2:** Perioperative outcomes.

Variable	Group 1 (*n* = 30)	Group 2 (*n* = 30)	*p* value
Operative time (min)	148.4 ± 26.1	204.7 ± 32.8	<0.001
Estimated blood loss (mL)	376.7 ± 114.3	653.3 ± 211.8	<0.001
Hospital stay (days)	3.7 ± 0.9	4.4 ± 0.8	0.004
Blood transfusion, *n* (%)	2 (6.7%)	8 (26.7%)	0.080
Dural tear, *n* (%)	1 (3.3%)	1 (3.3%)	1.000
Wound complication, *n* (%)	1 (3.3%)	1 (3.3%)	1.000
New neurological deficit, *n* (%)	0 (0.0%)	1 (3.3%)	1.000
Early reoperation ≤30 days, *n* (%)	0 (0.0%)	3 (10.0%)	0.237

Values are expressed as mean ± SD or *n* (%).

### ODI results

In Group 1, the mean preoperative ODI score was 50.6 ± 6.8, which improved to 32.5 ± 8.8 at postoperative month 1, 25.2 ± 8.7 at month 6, and 23.7 ± 8.5 at 2 years. In Group 2, ODI improved from 49.0 ± 8.2 preoperatively to 31.6 ± 8.4 at month 1, 22.9 ± 8.6 at month 6, and 19.8 ± 9.0 at 2 years. Within-group improvements were statistically significant at all time points in both groups (*p* < 0.001). ODI scores across follow-up are presented in Table 3.

**Table 3:** ODI scores across follow-up periods with within- and between-group comparisons.

Time point	Group 1 (*n* = 30)	p vs. preop (Group 1)^a^	Group 2 (*n* = 30)	p vs. preop (Group 2)^a^	*p* value (between-group)
Preoperative	50.6 ± 6.8	–	49.0 ± 8.2	–	0.417
1 month	32.5 ± 8.8	<0.001	31.6 ± 8.4	<0.001	0.686
6 months	25.2 ± 8.7	<0.001	22.9 ± 8.6	<0.001	0.287
2 years	23.7 ± 8.5	<0.001	19.8 ± 9.0	<0.001	0.087

Values expressed as mean ± SD.

a Within-group *p* values (Wilcoxon signed-rank test). Between-group comparisons performed using the Mann–Whitney U test. ODI, Oswestry Disability Index.

### VAS results

In Group 1, the VAS score decreased from 7.5 ± 0.9 preoperatively to 3.5 ± 1.2 at month 1, 2.4 ± 1.2 at month 6, and 2.2 ± 1.5 at 2 years. In Group 2, VAS decreased from 7.7 ± 1.0 to 4.2 ± 1.3 at month 1, 2.8 ± 1.3 at month 6, and 2.1 ± 1.4 at 2 years. Within-group improvements were statistically significant at all time points in both groups (*p* < 0.001). VAS scores are presented in Table 4.

**Table 4:** VAS pain scores across follow-up periods with within- and between-group comparisons.

Time point	Group 1 (*n* = 30)	p vs. preop (Group 1)^a^	Group 2 (*n* = 30)	p vs. preop (Group 2)^a^	*p* value (between-group)
Preoperative	7.5 ± 0.9	–	7.7 ± 1.0	–	0.551
1 month	3.5 ± 1.2	<0.001	4.2 ± 1.3	<0.001	0.022
6 months	2.4 ± 1.2	<0.001	2.8 ± 1.3	<0.001	0.250
2 years	2.2 ± 1.5	<0.001	2.1 ± 1.4	<0.001	0.784

Values expressed as mean ± SD.

a Within-group *p* values (Wilcoxon signed-rank test). Between-group comparisons performed using the Mann–Whitney U test. VAS, visual analogue scale.

### Intergroup comparison

No statistically significant differences were observed in ODI scores at postoperative month 1 (*p* = 0.686), month 6 (*p* = 0.287), or year 2 (*p* = 0.087). Regarding VAS, Group 1 demonstrated a significantly lower score at 1 month compared to Group 2 (3.5 ± 1.2 vs. 4.2 ± 1.3; *p* = 0.022), indicating a more favorable early pain recovery in the less invasive group. No significant between-group differences were observed in VAS at month 6 (*p* = 0.250) or year 2 (*p* = 0.784).

### Complications and revision surgery

Revision surgery at the L5–S1 level within 24 months was required in 2 patients (6.7%) in Group 1 and 2 patients (6.7%) in Group 2 (*p* = 1.000). In Group 1, one revision was performed for newly developed segmental instability and one for new L5–S1 disc herniation with radicular pain. In Group 2, two revisions were performed for symptomatic S1 screw loosening. Radiographic S1 screw loosening was identified in 7 of 30 patients (23.3%) in Group 2, of whom 2 (6.7%) were symptomatic and required revision. L5–S1 pseudarthrosis was observed in 2 patients (6.7%) in Group 2. Adjacent segment disease was identified in 2 patients (6.7%) in Group 1 and 3 patients (10.0%) in Group 2 (*p* = 1.000).

## Discussion

The lumbosacral junction represents one of the most biomechanically demanding regions of the spine due to the combination of high axial loading and substantial shear forces. Consequently, pedicle screw loosening at the S1 level remains a clinically significant complication following lumbosacral fusion procedures. Previous studies have reported that the incidence of S1 pedicle screw loosening ranges from 15.6% to 41.9% in the literature (1). Particularly in long-segment fusions, the position of the S1 screw as the distal endpoint of the construct increases the mechanical load on the screw, thereby elevating the risk of stress-related failure. In the present study, patients who underwent posterior lumbar stabilization and had radicular symptoms originating from the L5–S1 level were evaluated in the specific context of absent segmental instability, and two management strategies were compared.

A fundamental methodological consideration in this study is that all patients — in both groups — were confirmed to have no preoperative L5–S1 instability on flexion–extension radiographs. Patients with demonstrable instability were excluded from the outset, as sacral fixation is mandatory in such cases and renders comparison meaningless. This design ensures that the two groups are homogeneous in terms of their baseline biomechanical profile at the L5–S1 level, thereby mitigating the risk of selection bias that would arise if patients with different instability profiles were compared.

A key finding of this study is the significant difference in perioperative outcomes between the two groups. Group 1 demonstrated a mean operative time of 148.4 minutes compared to 204.7 minutes in Group 2 (*p* < 0.001), representing a reduction of approximately 56 minutes. Estimated blood loss was also substantially lower in Group 1 (376.7 vs. 653.3 mL; *p* < 0.001), as was the length of hospital stay (3.7 vs. 4.4 days; *p* = 0.004). These findings are consistent with the existing literature. Previous studies have reported that TLIF adds approximately 30–60 minutes of operative time and increases blood loss by 250–300 mL compared to non-interbody approaches (5, 6). Furthermore, the use of interbody techniques may necessitate neuromonitoring due to manipulation of neural structures, which in turn increases procedural costs and may negatively impact cost-effectiveness. The avoidance of these additional burdens, without compromise of clinical outcomes, represents a meaningful advantage of the selective decompression strategy.

During a two-year follow-up period, only two patients (6.67%) in Group 1 required revision surgery. One case was associated with disc progression, while the other demonstrated newly developed segmental instability. Notably, S1 pedicle screw placement was avoided in 93.33% of patients in Group 1 without adverse clinical consequences. In Group 2, radiographic S1 screw loosening was observed in 7 of 30 patients (23.3%), which falls within the range reported in the literature, and symptomatic loosening requiring revision occurred in 2 patients (6.7%).

The biomechanical behavior of sacral pedicle screws differs from that of lumbar levels. Biomechanical studies, particularly those conducted in osteoporotic models, have demonstrated that S1 screws are subjected to higher mechanical stress and that variations in insertion techniques can significantly influence load distribution (2). Furthermore, directing the S1 screw toward the apex of the sacral promontory has been reported to offer potential advantages compared with the conventional bicortical placement technique (3). However, the higher proportion of cancellous bone within the sacrum, the relatively shorter pedicle length, and the increased mechanical load at the lumbosacral junction are considered the principal factors contributing to an elevated risk of screw loosening.

In our study, both groups demonstrated significant improvements in ODI and VAS scores compared to preoperative values. This finding is consistent with the existing literature, which indicates that surgical treatment of lumbar degenerative pathologies has a positive impact on pain relief and functional outcomes. Previous studies have shown that both decompression and fusion procedures result in marked improvements in VAS and ODI scores, with the most pronounced recovery occurring within the first six months, followed by a plateau phase thereafter (8–10). Similarly, in our cohort, substantial early improvement was observed, and these favorable outcomes were maintained throughout the two-year follow-up period.

In the intergroup comparison, no statistically significant differences were observed in ODI scores at any time point. Regarding VAS, Group 1 demonstrated a statistically significant advantage at the 1-month follow-up (*p* = 0.022), which was no longer present at 6 months or 2 years. This early difference is likely attributable to the less extensive surgical dissection in the selective decompression group and is consistent with the known association between more invasive surgery and greater early postoperative pain. The absence of a significant difference at later time points suggests that the addition of TLIF and S1 fixation does not confer additional long-term clinical benefit in this patient population.

**Figure 6:**
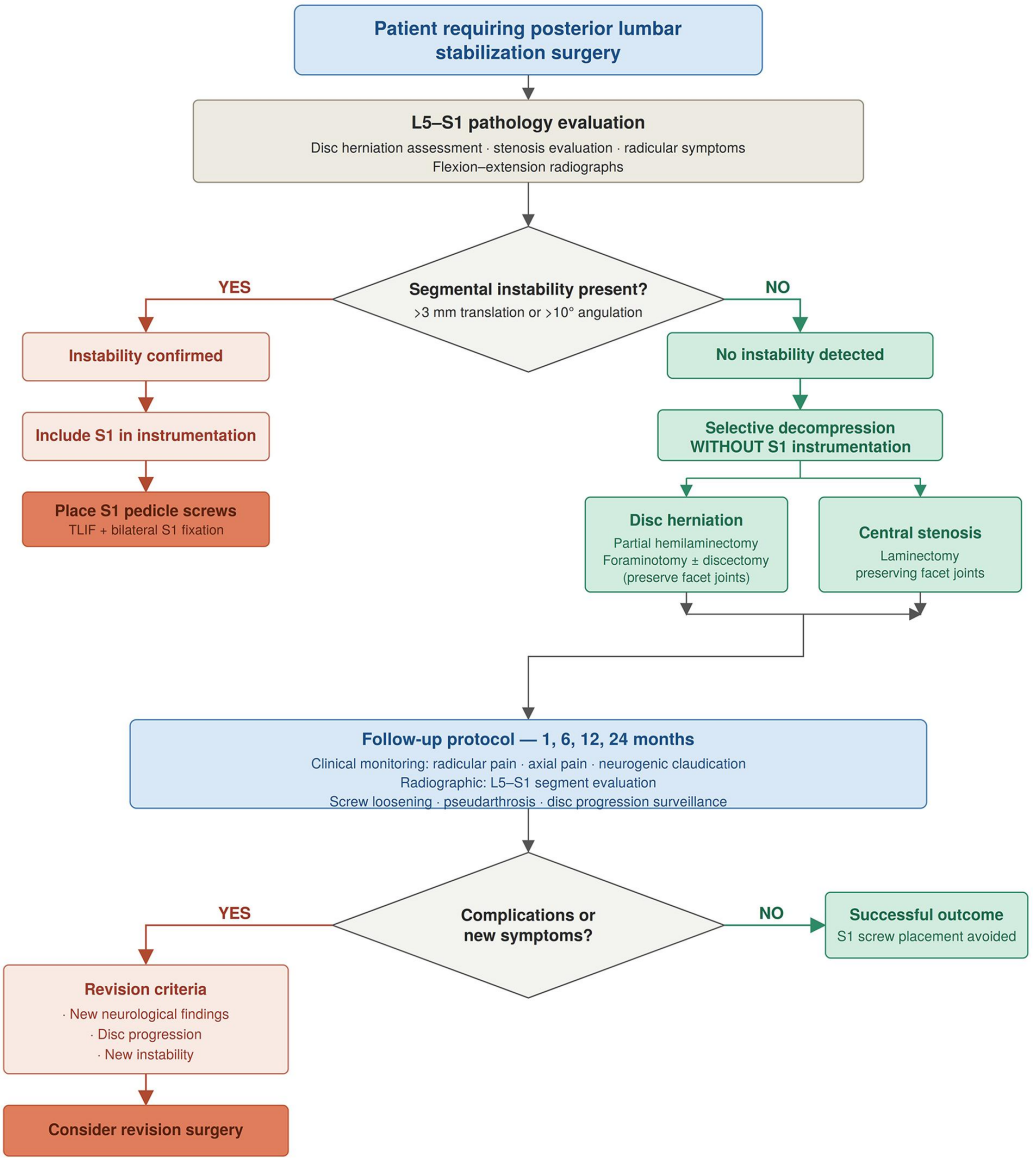
L5–S1 treatment decision-making algorithm in posterior lumbar stabilization surgery.

These findings suggest that, with careful preoperative and intraoperative assessment of segmental instability, inclusion of the L5–S1 level in the fusion construct may not be mandatory in all cases (Figure 6). Representative radiological examples are provided to illustrate the main surgical strategies and outcome patterns observed in this cohort, including successful L5–S1 preservation after selective decompression, late revision after recurrent L5–S1 pathology, S1 pedicle screw loosening, and stable TLIF with sacral fixation (Figures 1–4). In particular, in cases where no segmental motion is detected on flexion–extension radiographs, facet joint integrity is preserved, and posterior element stability remains intact, a selective decompression approach may represent a safe alternative. This strategy may offer potential advantages in terms of reducing surgical morbidity, shortening operative time, and lowering overall costs.

To the best of our knowledge, this study is among the limited number of investigations specifically evaluating the necessity of routinely including the L5–S1 level in the fusion construct in patients undergoing multi-level posterior stabilization without segmental instability at this level.

### Limitations

Our study has several limitations. The retrospective design and relatively small sample size limit the generalizability of the findings. Bone mineral density was not routinely recorded in all patients, which may influence screw loosening risk. The grouping was based on preoperative assessment of facet-preserving feasibility with intraoperative confirmation, rather than randomization, which may introduce residual confounding despite baseline homogeneity. Furthermore, the follow-up period may be considered mid-term. Comprehensive spinopelvic parameters beyond lumbar lordosis (including PI, PT, and SVA) were not systematically assessed, as the primary focus of this study was clinical and perioperative outcomes rather than sagittal alignment correction. Additional variables such as steroid use, Modic changes, and foraminal stenosis grade were not systematically recorded in this retrospective cohort and could not be included in the baseline analysis. Systematic radiographic measurements of disc height, foraminal height, and segmental lordosis at the L5–S1 level were not performed. Future prospective studies should incorporate standardized radiographic follow-up protocols and comprehensive imaging documentation. Future prospective, randomized studies with larger cohorts and longer follow-up durations are warranted to more clearly elucidate the long-term biomechanical and clinical outcomes of this approach.

## Conclusion

In this retrospective cohort of patients undergoing multi-level posterior lumbar fusion without L5–S1 instability, selective decompression of the L5–S1 segment without sacral instrumentation was associated with comparable mid-term clinical outcomes to TLIF with S1 pedicle screw fixation, while reducing operative time, blood loss, and S1 screw-related mechanical complications. These findings suggest that selective decompression may be considered as an alternative approach in carefully selected patients without radiographic L5–S1 instability. Prospective randomized studies with larger cohorts are warranted to confirm these preliminary findings.

## Data Availability

The raw data supporting the conclusions of this article will be made available by the authors, without undue reservation.
